# Protective Effects of Deferoxamine on Vestibulotoxicity in Gentamicin-Induced Bilateral Vestibulopathy Rat Model

**DOI:** 10.3389/fneur.2021.650752

**Published:** 2021-03-24

**Authors:** Hyo-Jung Kim, Jin-Ok Lee, Ji-Soo Kim

**Affiliations:** ^1^Research Administration Team, Seoul National University Bundang Hospital, Seongnam, South Korea; ^2^Department of Biomedicine and Health Sciences, Graduate School, The Catholic University of Korea, Seoul, South Korea; ^3^Department of Neurology, Seoul National University College of Medicine, Seoul, South Korea; ^4^Department of Neurology, Dizziness Center, Clinical Neuroscience Center, Seoul National University Bundang Hospital, Seongnam, South Korea

**Keywords:** deferoxamine, iron-chelating agent, ototoxicity, rats, dizziness, bilateral vestibulopathy

## Abstract

**Introduction:** Administration of aminoglycoside (AG) antibiotics is one of the most common causes of ototoxicity. This study aimed to determine the protective effects of deferoxamine, an iron-chelating agent, on vestibulotoxicity using an intratympanic gentamicin injection (ITGM)-induced bilateral vestibulopathy rat model.

**Methods:** Fifteen Sprague-Dawley rats were randomly assigned to the ITGM only (*n* = 5), the ITGM combined with intramuscular deferoxamine (DFO) injection (ITGM+DFO, *n* = 5), or the intratympanic normal saline (control, *n* = 5) group. The rats in the ITGM+DFO group received intramuscular injection of 150 mg/kg of deferoxamine at 30, 90, and 150 min after the ITGM. The vestibular function was evaluated using the rotarod and open field test every 3 days after the injection until Day 16 when the rats were subjected to histological changes.

**Results:** The rats in the ITGM only group began to show significantly impaired vestibular function 2 days after ITGM into both ears. In contrast, the vestibular function was maintained in the control and ITGM+DFO groups without a difference throughout the experiments. The rats in the ITGM only group showed a near-complete loss of the type I and II hair cells and a collapse of the sensory epithelium in both the saccule and utricle. In contrast, the rats in the ITGM+DFO and control groups showed a relatively well-preserved sensory epithelium including the hair cells, cilia, and otolith layer.

**Conclusion:** This study provides experimental evidence for preventive effects of iron-chelating agents on AG-induced vestibulotoxicity. Simultaneous administration of iron-chelating agents may be considered when using ototoxic agents, especially in those considered to be vulnerable to toxic damage of the inner ear.

## Introduction

Ototoxicity accounts for a significant proportion of audiovestibular loss worldwide (1). Numerous drugs and chemical substances are known to affect the cochlea, vestibule, or both (1). Bilateral vestibulopathy is a disabling disorder characterized by dizziness, oscillopsia, and unsteadiness mostly during locomotion due to hypofunction of the vestibular organs on both sides (2–5). The most common etiology of bilateral vestibulopathy is ototoxicity due to aminoglycoside (AG) antibiotics, followed by Meniere's disease, infectious diseases, and genetic disorders (5). Since the vestibular hair cells do not regenerate in humans (6), prevention is the most important when expected, and managements of bilateral vestibulopathy mostly concentrate on central adaptation and sensory substitution when it occurs (7).

Since the AG-induced ototoxicity starts from a formation of AG–iron complex (8), depletion of iron using deferoxamine, an iron-chelating agent, may prevent or reduce the inner ear damage induced by AG administration. Furthermore, deferoxamine is known to have a role in scavenging the reactive oxygen species (ROS) that are involved in apoptosis of the neural cells (8). Previous studies, however, have mostly focused on the preventive effects of deferoxamine on cochleotoxicity induced by AG (9, 10). The aim of this study was to determine the protective effects of deferoxamine on vestibulotoxicity using an intratympanic gentamicin injection (ITGM)-induced bilateral vestibulopathy rat model.

## Methods

### Materials

We used 15 Sprague-Dawley rats weighing 180–220 g with a normal Preyer's reflex. This study was approved by the Institutional Animal Care and Use Committee of Seoul National University Bundang Hospital (IACUC no. BA-2003-293-035-01).

### Study Design

**Figure 1:**
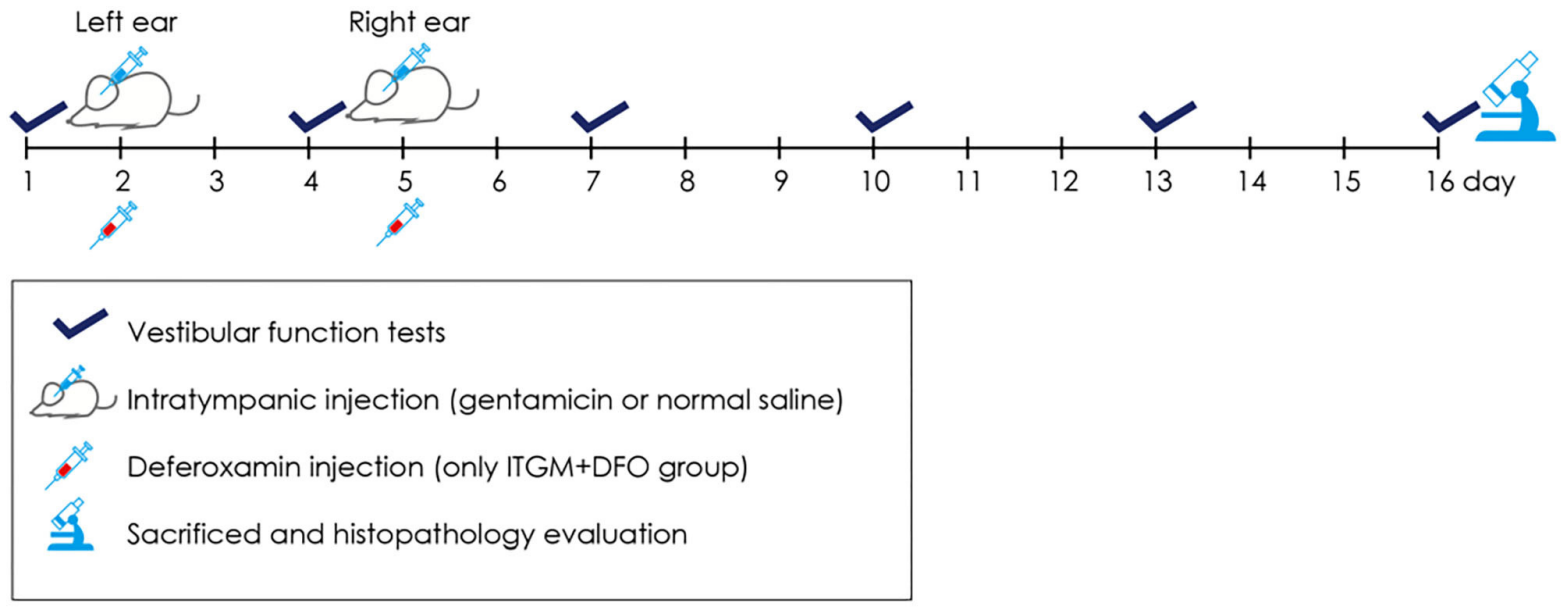
A schematic diagram describing the experimental procedure. Every 3 days from Day 1 until Day 16, the animals had evaluation of the vestibular function using the rotarod and open field tests. On Day 2, each rat received the intratympanic gentamicin injection (ITGM) in the ITGM only and ITGM + intramuscular deferoxamine (DFO) injection groups or intratympanic normal saline injection (ITNS) in the control group into the left ear. On Day 5, the rats had ITGM or ITNS into the right ear. The rats in the ITGM+DFO group received intramuscular injection of deferoxamine at 30, 90, and 150 min after the ITGM. On Day 16, the rats were sacrificed for histological studies.

The rats were randomly assigned to the ITGM only (*n* = 5), the ITGM combined with intramuscular deferoxamine (DFO) injection (ITGM+DFO, *n* = 5), or the control (*n* = 5) group. The experimental procedure is illustrated in the Figure 1. On Day 1, all the rats had a baseline evaluation of the vestibular function using the rotarod and open field tests. On Day 2, each rat received ITGM in the ITGM only and ITGM+DFO groups or intratympanic normal saline injection (ITNS) in the control group into the left ear. The volume of intratympanic injections was ~0.2 ml, and a gelform soaked with GM or normal saline had been placed at the tympanic membrane for 3 days thereafter. On Day 5, the rats had ITGM or ITNS into the right ear after having removed the gelform from the left ear and having confirmed a needle hole on the tympanic membrane from the previous injection. The rats in the ITGM+DFO group received intramuscular injection of 150 mg/kg of deferoxamine (desferrioxamine mesylate, Hospira Australia Pty Ltd., Australia) at 30, 90, and 150 min after the ITGM. Evaluation of the vestibular function was repeated every 3 days until Day 16 (six trials) using the rotarod and open field tests. The rats were then sacrificed to determine any histological changes.

### Rotarod Test

The rotarod test was developed to evaluate the balance function and activity of animals on the rotating drum (Rota-Rod, Jeung Do Bio & Plant Co., Ltd., Korea). The experiment started from positioning of each rat on the rotarod drum. We measured the time until the rats fell off the drum. Based on the results of preliminary trials having adopted the rotating velocities varying from 5 to 40 RPMs, we decided to set the RPM at 10 during the experiments since this RPM could determine the rats' balance function best. If the rat did not fall off for more than 3 min in each trial, the experiment was terminated. For each trial, the latency to fall from the rotating rod was measured.

### Open Field Test

The open field test was developed to assess general locomotor activity and anxiety level in the animals. We used a box of 60 × 60 × 40 cm in size. The behavior of each rat was recorded for 5 min using a SMART 3.0 video-tracking system (Harvard Apparatus, USA). And then, the positions of the rats in the recorded video clips were plotted on the coordinate in real-time manner. Total moving distance and resting time were measured using the SMART 3.0 analysis program.

### Histopathology

On Day 16, rats were sacrificed under anesthesia using inhalation of 2% isoflurane. After exposing the heart, we made a small incision through the apex of the left ventricle to insert the perfusion catheter and another small incision in the right auricle to let the perfusate escape. Subsequent to transcardiac perfusion with 200 ml of normal saline for exsanguination, the rats were perfused with 300 ml of 4% formaldehyde. The temporal bones containing the inner ear were dissected out and then immersed in the 4% formaldehyde overnight at room temperature. The fixed tissues were again immersed in Calci-clear Rapid (hydrochloric acid, EDTA; National Diagnostics, Atlanta, GA) for 12 h before the processing for paraffin blocks, which included dehydration, clearing, infiltration, and embedding. The blocks were then sectioned with a 3-μm thickness and subjected to hematoxylin and eosin (H&E) staining.

### Statistical Analyses

The latency during the rotarod test and the total moving distance and resting time during the open field test were compared among the groups using the linear mixed model. We defined the groups (ITGM only, ITGM+DFO, and ITNS) and experiment dates (Days 1, 4, 7, 10, 13, and 16) as the fixed factors and the individual rats as a random factor for the linear mixed model. The rotarod and open field tests (total moving distance and resting time) were repeatedly measured every 3 days until Day 16. For *post-hoc* analyses, pairwise comparisons were performed based on estimated marginal means. The degrees of freedom was defined using the Kenward–Roger method, and the *p-*values were adjusted using the Tukey method. All the tests were performed using R (version 3.3.3, https://www.r-project.org/), and *p* < 0.05 was considered significant.

## Results

### Rotarod Test

**Figure 2:**
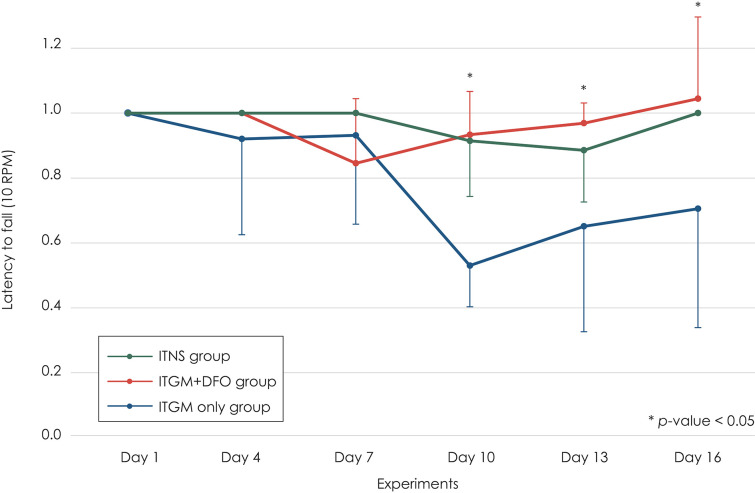
The latency to fall from the rotarod drum at 10 RPM. The rats in the intratympanic gentamicin injection (ITGM) only group showed a marked drop in the latency, which persisted until the end of experiments with a significant difference from that in the ITGM + intramuscular deferoxamine (DFO) and intratympanic normal saline injection (ITNS) groups on Day 10 [pairwise comparisons, ITGM only vs. ITGM+DFO: t (88.1) = 3.130, *p* = 0.007, ITGM only vs. ITNS: t (88.1) = −2.983, *p* = 0.010]. On Days 14 and 16, the difference was significant only between the ITGM only and ITGM+DFO groups [pairwise comparisons, Day 14: t (88.1) = 2.467, *p* = 0.04, Day 16: t (88.1) = 2.467, *p* = 0.027]. In contrast, the latency did not differ between the ITNS and ITGM+DFO groups throughout the experiments. The latency obtained during the follow-up evaluation was normalized to the baseline value due to a wide individual variation. The data are expressed as a mean ± standard deviation.

At the baseline (Day 1), most rats did not fall from the drum for 3 min. This performance had been maintained until 2 days after the second injection (Day 7). After then, the rats in the ITGM group showed a marked drop in the latency [linear mixed model, F (10, 75) = 2.319, *p* = 0.019], which persisted until the end of experiments. On Day 10, the difference was statistically significant between the ITGM only and ITGM+DFO groups [pairwise comparisons, t (88.1) = 3.130, *p* = 0.007] and between the ITGM only and control (ITNS) groups [pairwise comparisons, t (88.1) = −2.983, *p* = 0.010]. On Days 14 and 16, the significant difference was found only between the ITGM only and ITGM+DFO groups [pairwise comparisons, Day 14: t (88.1) = 2.467, *p* = 0.04, Day 16: t (88.1) = 2.467, *p* = 0.027]. In contrast, the latency did not differ between the ITGM+DFO and control groups throughout the experiments (Figure 2).

### Open Field Test

**Figure 3:**
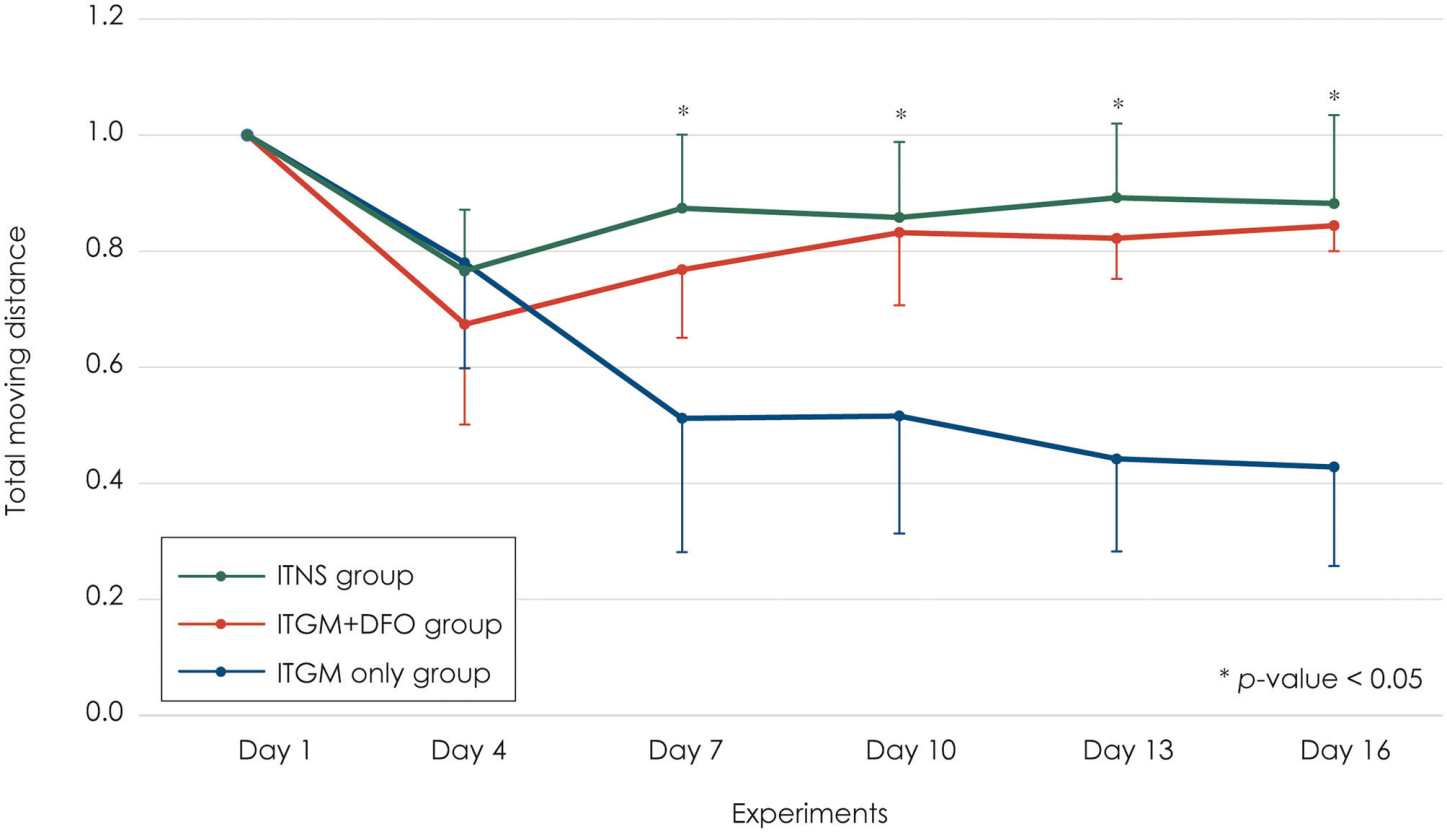
The total moving distance. The rats in the intratympanic gentamicin injection (ITGM) only group showed a further decrease in the total moving distance 2 days after the second injection with a gradual decline thereafter [linear mixed model, F (10, 75) = 6.079, *p* < 0.001]. Thus, the total moving distance significantly decreased more in the ITGM only group than in the ITGM + intramuscular deferoxamine (DFO) and intratympanic normal saline injection (ITNS) groups from Day 7, 2 days after the second injection [pairwise comparisons, Day 7: t (77.5) = 2.667, *p* = 0.025, Day 10: t (77.5) = 3.292, *p* = 0.004, Day 14: t (77.5) = 3.958, *p* < 0.001, Day 16: t (77.5) = 4.333, *p* < 0.001]. The values obtained during the follow-up evaluation were normalized to the baseline ones due to a wide individual variation. The data are expressed as a mean ± standard deviation.

At the baseline (Day 1), the rats in all three study groups moved actively across the whole field. Two days after the first injection into the left ear (Day 4), the rats in all three groups showed a mild decrease (20–30%) in the total moving distance without a difference among the groups (Figure 3). Two days after the second injection into the contralateral right ear (Day 7), the rats in the control and ITGM+DFO groups showed no changes in the total moving distance, and the performance had been maintained until the last evaluation on Day 16 without a difference between the groups. In contrast, the rats in the ITGM only group showed a further decrease in the total moving distance 2 days after the second injection (Day 7) with a gradual decline thereafter [linear mixed model, F (10, 75) = 6.079, *p* < 0.001]. Thus, the total moving distance decreased more in the ITGM only group than in the control and ITGM+DFO groups from Day 7, 2 days after the second injection [pairwise comparisons, Day 7: t (77.5) = 2.667, *p* = 0.025, Day 10: t (77.5) = 3.292, *p* = 0.004, Day 14: t (77.5) = 3.958, *p* < 0.001, Day 16: t (77.5) = 4.333, *p* < 0.001; Figure 3, Supplementary Video 1].

**Figure 4:**
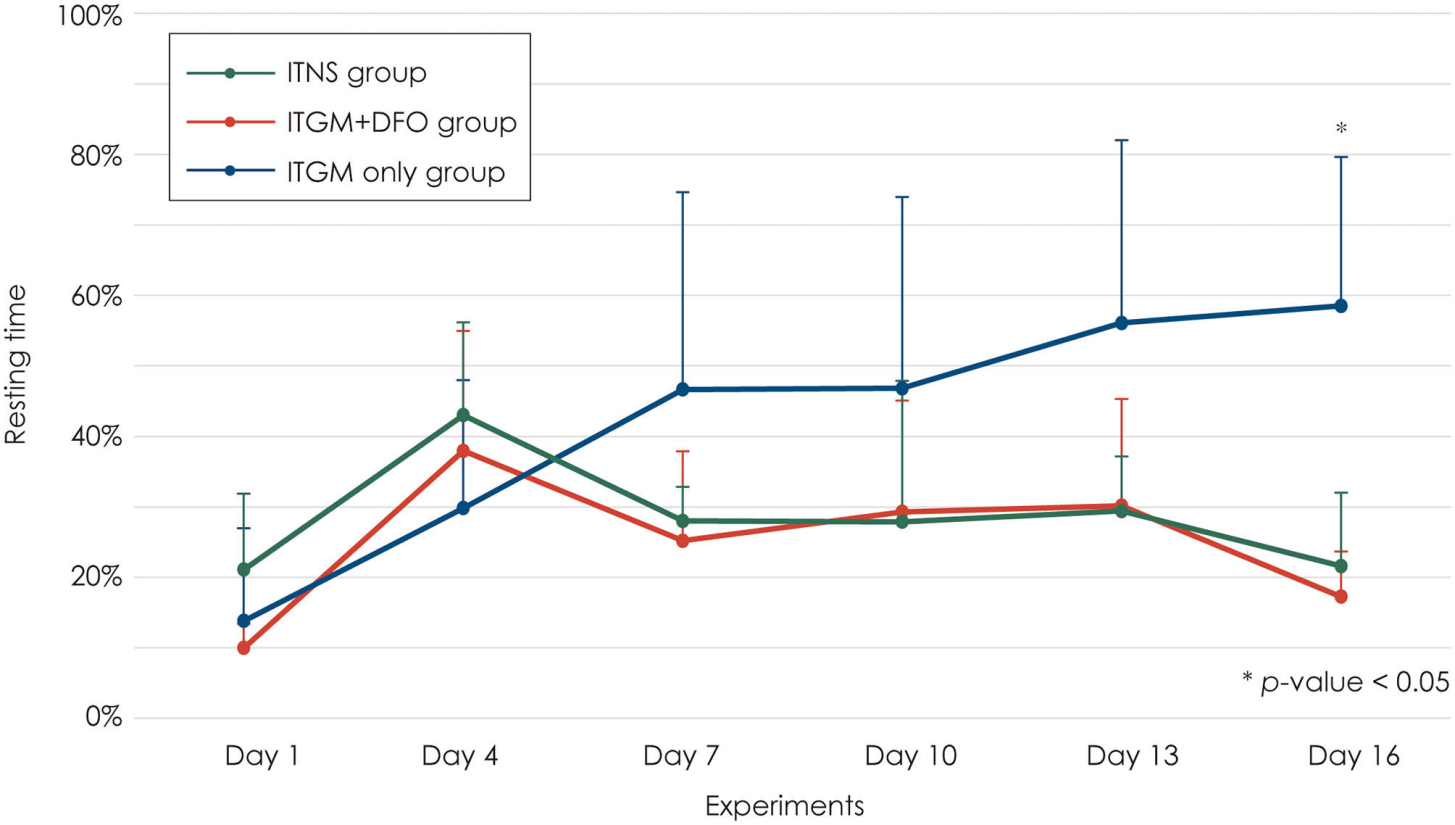
Resting time. The rats in the intratympanic gentamicin injection (ITGM) group showed a further increase in the resting time on Day 7 with a gradual increase thereafter with a statistically significant difference on Day 16 [linear mixed model, F (10, 75) = 4.406, *p* < 0.001; pairwise comparisons, ITGM only vs. ITGM+DFO: t (47.7) = −3.498, *p* = 0.003, ITGM only vs. ITNS: t (47.7) = 3.130, *p* = 0.008]. The data are expressed as a mean ± standard deviation. ITGM+DFO, ITGM + intramuscular deferoxamine; ITNS, intratympanic normal saline injection.

The resting time increased 2 days after the first injection (Day 4) in all three groups without a difference among the groups [linear mixed model, F (10, 75) = 4.406, *p* < 0.001; Figure 4]. Two days after the second injection (Day 7), the rats in the control and ITGM+DFO groups showed a mild decrease in the resting time without a difference between the groups, which had been generally maintained until the last evaluation. In contrast, the rats in the ITGM only group showed a further increase in the resting time on Day 7 with a gradual increase thereafter. However, the difference in the resting time was statistically significant only on Day 16 [pairwise comparisons, ITGM only vs. ITGM+DFO: t (47.7) = −3.498, *p* = 0.003, ITGM only vs. ITNS: t (47.7) = 3.130, *p* = 0.008; Figure 4, Supplementary Video 1].

### Histopathology

**Figure 5:**
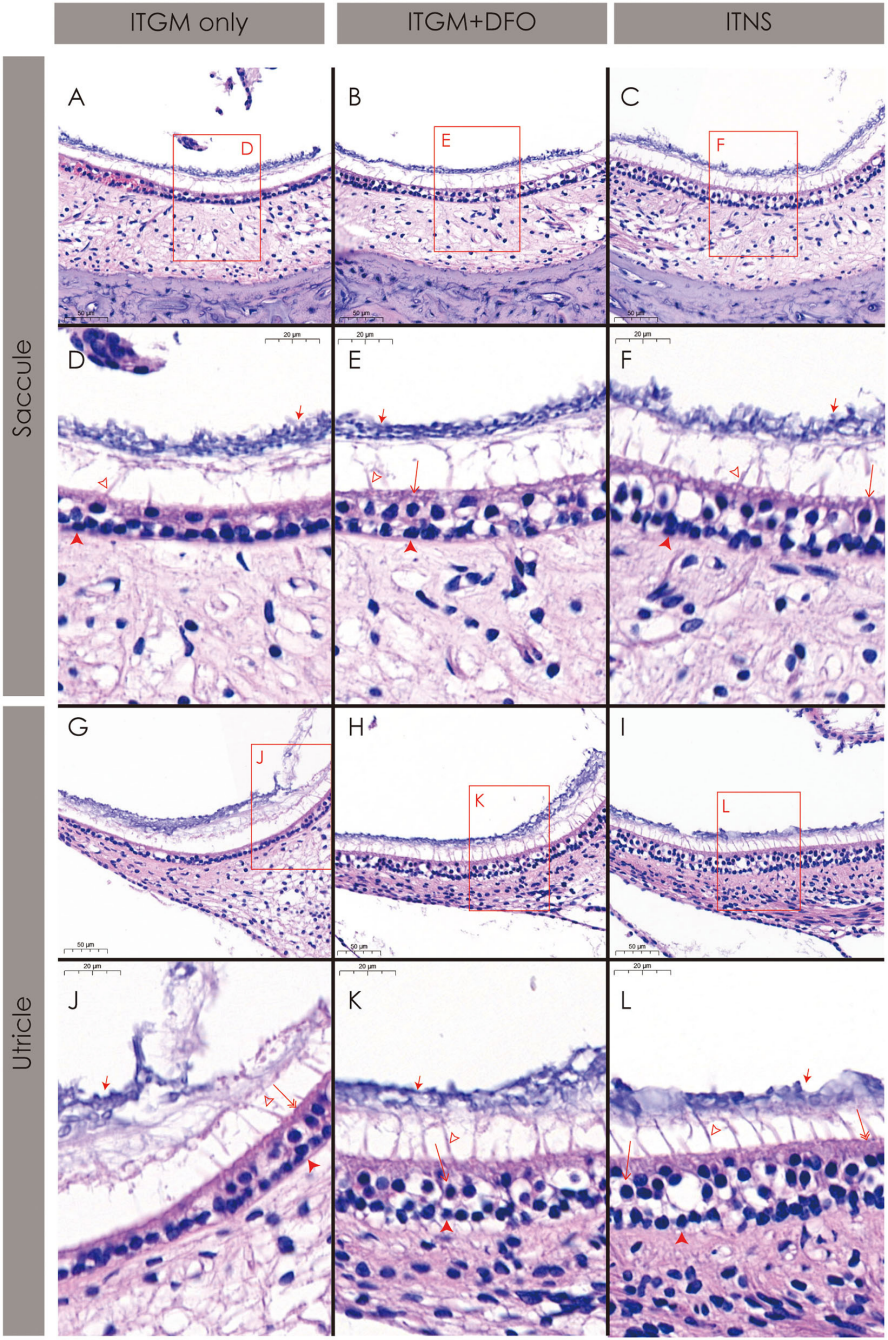
Histopathology of the inner ear. The rats in the intratympanic gentamicin injection (ITGM) only group show a near-complete loss of both the type I and II hair cells and a collapse of the sensory epithelium both in the saccule **(A,D)** and utricle **(G,J)**. In contrast, the rats in the ITGM combined with intramuscular deferoxamine (DFO) injection (ITGM+DFO) and normal control (ITNS) groups showed a relatively well-preserved sensory epithelium including the hair cells, cilia, and otolith layer in both the saccule **(B,C,E,F)** and utricle **(H,I,K,L)**. Open arrow, the type I hair cell; double open arrow, the type II hair cell; closed arrow, the otoconia; filled arrow head, the supporting cell; lined arrow head, the cilia.

The rats in the ITGM only group showed a near-complete loss of both the type I and II hair cells and a collapse of the sensory epithelium in both the saccule (Figures 5A,D) and utricle (Figures 5G,J). Even in the relatively preserved portions, the distinction between the hair types was impossible, and the cilia and otoconia layer were also separated from the sensory epithelium and became indistinct (Figures 5A,D,G,J).

In contrast, the rats in the ITGM+DFO and normal control groups showed a relatively well-preserved sensory epithelium including the hair cells, cilia, and otolith layer in both the saccule (Figures 5B,C,E,F) and utricle (Figures 5H,I,K,L).

## Discussion

This study documented preservation of the vestibular function and morphology in rats with ITGM when deferoxamine was co-administered. Indeed, the behavioral and morphological changes observed after the injections did not differ between the ITNS (control) and ITGM+DFO groups, while the ITGM only group showed a significant loss of the vestibular hair cells and a decrease in the activity after the injections. These indicate a protective effect of deferoxamine on AG-induced vestibulotoxicity.

### Study Design and Findings

We used ITGM to induce bilateral vestibulopathy in this study. Among the AG, gentamicin (GM) and streptomycin are the two most common vestibulotoxic drugs. Indeed, these drugs have been used for chemical ablation of the inner ear in Meniere's disease (11, 12) and other disorders (13–15). We adopted the ITGM rat model since it is a well-established method of inducing bilateral vestibulopathy in our laboratory (16). Indeed, the rats in the ITGM only group in our study showed the characteristic patterns of behavior and pathology that had been observed in AG-induced bilateral vestibulopathy. Previous studies on GM-induced ototoxicity in humans also showed a loss and vacuolization of the hair cells in both the semicircular cristae and otolithic organs with a relative sparing of the cochlear labyrinth (17, 18). In general, the type I hair cells are known to be more sensitive to the AG-induced vestibulotoxicity than the type II hair cells (19).

Subcutaneous injection of GM attains a peak in the serum level within 1 h and the trough within 6 h (20). In the perilymph, the peak is reached within 3–6 h with a near-complete disappearance within 24–36 h (20). When administered through the tympanic membrane, the peak level of GM in the perilymph is attained within 56 ± 21 min with a half-life of 117 ± 47 min (21). Since the plasma deferoxamine concentration reaches a peak 30 min after intramuscular injection with a half-life of only 1 h (22), we administered deferoxamine three times, at 30, 90, and 150 min after ITGM, to maintain a stable plasma concentration around the peak perilymph concentration of GM even though the pharmacokinetics of deferoxamine is unknown in the perilymph. Thus, to ensure the maximal protective effect of deferoxamine on the ototoxicity induced by GM, the dosing schedule of deferoxamine should be adjusted according to the route of administration of GM.

### The Mechanism of Aminoglycoside-Induced Ototoxicity

AG including GM is bactericidal against gram-negative aerobes. AG enters the hair cells and combines with iron to form an AG–iron complex (8). This AG–iron complex then reacts with the electron donors, such as arachidonic acids, to generate ROS. The ROS then stimulates the c-Jun N-terminal kinase, and the activated c-Jun N-terminal kinase is translocated into the nucleus to activate the genes involved in the cell death pathway (8, 23). These genes in turn release cytochrome *c* (cyt *c*), one of the proapoptotic proteins in the mitochondria (24). When released into the cytosol, the cyt *c* may interact with a protein called Apaf-1 (25). This interaction leads to recruitment of pro-caspase 9 into the multiprotein complex with cyt *c* and Apaf-1, which is called the apoptosome (25). Caspase proteins are one of the main executors of the apoptotic process, and formation of the apoptosome leads to activation of caspase-induced apoptosis (8, 25).

Otherwise, the ototoxicity induced by AG may involve activation of the N-methyl-d-aspartate (NMDA) receptors (26, 27). Based on this, a previous study showed a protective effect of memantine in amikacin-induced ototoxicity in a rabbit model (28).

### Protective Effects of Deferoxamine on Ototoxicity

In this study, administration of deferoxamine just after ITGM decreased the vestibulotoxicity as was determined with behavioral and histological changes in rats. This protective effect may have been achieved by chelating iron in the serum and thus decreasing formation of the AG–iron complex. Otherwise, this protective effect may also be ascribed to the role of deferoxamine as a ROS scavenger. Indeed, deferoxamine protected against bleb formation and cell length decrease induced by the ROS in isolated cochlear outer hair cells (29). Deferoxamine also decreased the shifts in the threshold of compound action potential in the cochlea exposed to ROS (30).

Likewise AG, cisplatin, a widely used chemotherapeutic agent to treat malignant tumors, also commonly shows ototoxicity, nephrotoxicity, and neurotoxicity (31). The mechanism of cisplatin-induced hair cell death is similar to that of the AG-induced ototoxicity. However, cisplatin is hydrated to form the monohydrate complex (MHC) after entering the hair cells. Then, the cisplatin-MHC activates the NOX-3, which would result in ROS production (8). The remaining process leading to cell death would be same as that in AG-induced ototoxicity (8). Other ototoxic agents including certain UV radiation, loop diuretics, antimalarial agents, and metals are known to generate ROS and thus hair cell loss (30). Thus, deferoxamine may exert a protective effect on ototoxicity induced by these substances.

### Clinical Implications

The individuals particularly at higher risk for ototoxicity include the elderly, those with multisystem disease, and those receiving multiple ototoxic medications (1). Since AG is predominantly excreted via the kidney through glomerular filtration, patients with renal failure may be at higher risk for accumulating AG in the blood and inner ear, causing further ototoxicity. Furthermore, AG-induced ototoxicity may be enhanced by simultaneous administration of loop diuretics (32). Familial or genetic predisposition has been established in AG-induced ototoxicity (33–35) with a probable autosomal dominant or X-linked or mitochondrial transmission. A previous study also found a mitochondrial nucleotide 1,555 A to G substitution in the 12S rRNA gene, which is known to affect the AG activity (36). Thus, administration of iron-chelating agents or free radical scavengers should be considered when the medication of ototoxic drugs is inevitable, especially in those vulnerable to ototoxicity.

Since deferoxamine itself may exert a toxic effect on the auditory and visual system, however, the benefits of deferoxamine should be weighed against its toxic effects (37). The ototoxicity of deferoxamine appears to be related to individual susceptibility and the dose administered. However, deferoxamine at doses lower than 50 mg/kg/day has been reported to be safe for eyes even though it may be slightly toxic to ears (37, 38). In this study, we used 450 mg/kg of deferoxamine over 2 h, which corresponds to human equivalent dose of 73 mg/kg. Given the short half-life of deferoxamine and duration of the follow-ups after the deferoxamine administration, the deferoxamine toxicity should have been negligible, if any, based on the near-complete preventive effects of deferoxamine on vestibulotoxicity induced by ITGM in the rats. It may have been related to the short duration of deferoxamine administration or any species-specific vulnerability to the deferoxamine toxicity. The optimal dose and duration of administration of deferoxamine, however, should be validated further in humans.

### Limitations

This study has several limitations. First, we were not able to determine the morphology and function of the semicircular canals. This was primarily due to technical limitations of our animal laboratory, and further studies should adopt quantitative evaluation of the rotational vestibulo-ocular reflex and morphologic assessment of the crista ampullaris. Second, the morphological assessment was unblinded and only qualitative. Thus, the histological findings would have been subjective and were inevitably subjected to a bias. Third, we adopted an ITGM rat model to induce vestibular ototoxicity. Since the ototoxicity of AG is mostly induced by systemic administration in humans, the animal model adopted in this study may not represent a real-world clinical setting. Finally, the small sample size adopted in this study may limit the generalizability of the results given the huge individual variations in animals' behavior.

## Conclusion

Awareness, monitoring, and appropriate care including preventive therapy are important in reducing ototoxicity. This study provides experimental evidence for the protective effects of deferoxamine on AG-induced vestibulotoxicity. Simultaneous administration of iron chelating agents or ROS scavengers may be attempted when using ototoxic agents, especially in those considered to be vulnerable to ototoxicity.

## Data Availability Statement

The raw data supporting the conclusions of this article will be made available by the authors, without undue reservation.

## Ethics Statement

The animal study was reviewed and approved by Institutional Animal Care and Use Committee of Seoul National University Bundang Hospital.

## Author Contributions

H-JK acquired and analyzed the data and drafted the manuscript. J-OL acquired and analyzed data. J-SK conducted the design and conceptualization of the study, interpretation of the data, and revision of the manuscript. All authors contributed to the article and approved the submitted version.

## Conflict of Interest

The authors declare that the research was conducted in the absence of any commercial or financial relationships that could be construed as a potential conflict of interest.

## Supplementary Material

Supplementary Video 1 The activity of the rats during the open filed test. The left side of the screen is the baseline (Day 1), and the right side is from Day 16. The rats in the intratympanic gentamicin injection (ITGM) only group showed a markedly reduced activity and crouch in the corner. In contrast, the rats in the ITGM combined with intramuscular deferoxamine (DFO) injection (ITGM+DFO) and normal control (ITNS) groups show an activity similar to that at the baseline. The video is played at an 8 × speed.
